# Mitochondria in cancer: a comprehensive review, bibliometric analysis, and future perspectives

**DOI:** 10.1007/s12672-025-02139-5

**Published:** 2025-04-11

**Authors:** Abeer El Wakil, Patrick Devos, Heba Abdelmegeed, Alaa Kamel

**Affiliations:** 1 Department of Biological and Geological Sciences, Faculty of Education, Alexandria University, Alexandria, 21526 Egypt; 2 Université Lille, Lillometrics, 59000 Lille, France; 3 CHU Lille, Direction de la Recherche et de l’Innovation, 59000 Lille, France; 4 Department of Chemistry of Natural Compounds, National Research Centre, Giza, Egypt; 5 Department of Zoology, Faulty of Science, Alexandria University, Alexandria, Egypt

**Keywords:** Mitochondria, Neoplasm, Liver, Breast, Lung, Bibliometrics

## Abstract

**Introduction:**

Mitochondria are essential organelles for many aspects of cellular homeostasis. They play an indispensable role in the development and progression of diseases, particularly cancer which is a major cause of death worldwide. We analyzed the scientific research output on mitochondria and cancer via PubMed and Web of Science over the period 1990–2023.

**Methods:**

Bibliometric analysis was performed by extracting data linking mitochondria to cancer pathogenesis over the period 1990–2023 from the PubMed database which has a precise and specific search engine. Only articles and reviews were considered. Since PubMed does not support analyses by countries or institutions, we utilized InCites, an analytical tool developed and marketed by Clarivate Analytics. We also used the VOSviewer software developed by the Centre for Science and Technology Studies (Bibliometric Department of Leiden University, Leiden, Netherlands), which enables us to graphically represent links between countries, authors or keywords in cluster form. Finally, we used iCite, a tool developed by the NIH (USA) to access a dashboard of bibliometrics for papers associated with a portfolio. This module can therefore be used to measure whether the research carried out is still basic, translational or clinical.

**Results:**

In total, 169,555 publications were identified in PubMed relating to ‘mitochondria’, of which 34,949 (20.61%) concerned ‘mitochondria’ and ‘dysfunction’ and 22,406 (13.21%) regarded ‘mitochondria’ and ‘cancer’. Hence, not all mitochondrial dysfunctions may lead to cancer or enhance its progression. Qualitatively, the disciplines of journals were classified into 166 categories among which cancer specialty accounts for only 4.7% of publications. Quantitatively, our analysis showed that cancer/neoplasms in the liver (2569 articles) were placed in the first position. USA occupied the first position among countries contributing the highest number of publications (5695 articles), whereas Egypt came in the thirty-eight position with 84 publications (0.46%). Importantly, USA is the first-ranked country having both the top 1% and 10% impact indicators with 207 and 1459 articles, respectively. By crossing the query ‘liver neoplasms’ (155,678) with the query ‘mitochondria’ (169,555), we identified 1336 articles in PubMed over the study period. Among these publications, research areas were classified into 65 categories with the highest percentage of documents included in biochemistry and molecular biology (28.92%), followed by oncology (23.31%).

**Conclusions:**

This study underscores the crucial yet underrepresented role of mitochondria in cancer research. Despite their significance in cancer pathogenesis, the proportion of related publications remains relatively low. Our findings highlight the need for further research to deepen our understanding of mitochondrial mechanisms in cancer, which could pave the way for new therapeutic strategies.

**Graphical Abstract:**

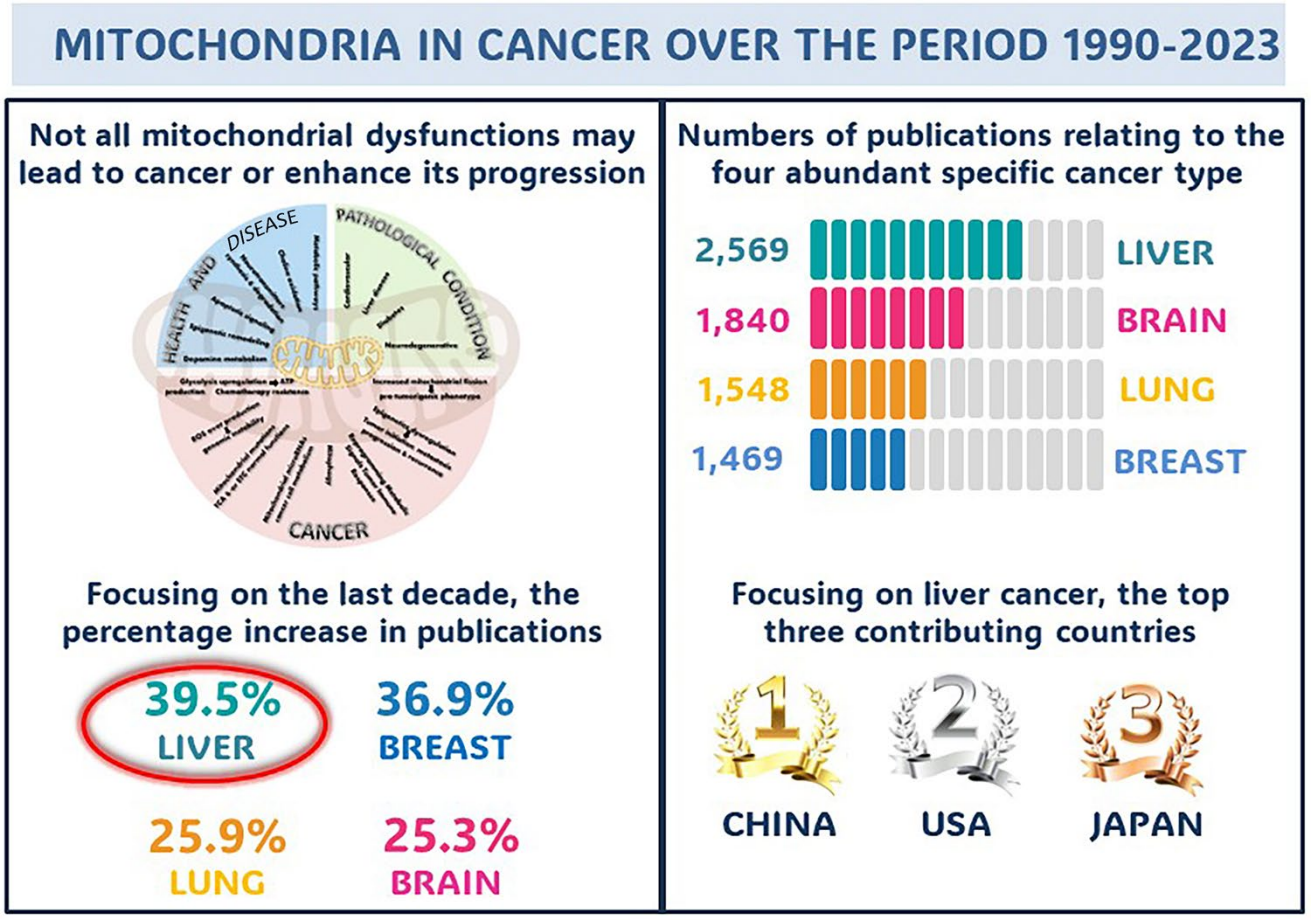

**Supplementary Information:**

The online version contains supplementary material available at 10.1007/s12672-025-02139-5.

## Introduction

Mitochondria are indispensable cellular organelles with a unique structure essential for maintaining cellular homeostasis. They consist of two phospholipid membranes, the outer mitochondrial membrane (OMM) and the inner mitochondrial membrane (IMM), which enclose the matrix and the intermembrane space [1]. These membranes differ in lipid composition, shape, permeability and embedded transmembrane proteins. The OMM proteins regulate mitochondrial import systems, fusion, and fission, while the IMM primarily houses.

respiratory chain complexes responsible for oxidative phosphorylation (OXPHOS) and ATP (adenosine triphosphate) synthesis [2]. The IMM is further divided into the inner boundary membrane and the cristae, where respiratory chain complexes are densely packed to optimize ATP production [1]. This intricate structure is crucial for supporting vital cellular functions, particularly energy metabolism and cell signaling.

Beyond their well-established role in ATP generation [3], mitochondria play a central role in regulating cell fate through apoptotic signaling, reactive oxygen species (ROS) production, and metabolic adaptation. Mitochondrial dysfunction is increasingly recognized as a hallmark of cancer, contributing to tumor initiation, progression, metastasis, and resistance to therapy. Altered mitochondrial metabolism supports the metabolic reprogramming of cancer cells, enabling them to meet the high bioenergetic and biosynthetic demands of rapid proliferation. Additionally, mitochondrial dynamics, including fusion, fission, and mitophagy, modulate cellular stress responses and influence the tumor microenvironment. Understanding and manipulating these processes offer promising therapeutic avenues for combating mitochondrial-related malignancies.

Cancer remains a leading cause of mortality and a significant barrier to increasing life expectancy worldwide [4]. According to World Health Organization (WHO) estimates from 2019 [5], cancer ranks as the first or second leading cause of death before the age of 70 in 112 of 183 countries and third to fourth in an additional 23 countries. The rising global cancer burden is driven by an aging population and increased exposure to risk factors, necessitating urgent research efforts to develop effective treatment strategies [4]. The growing recognition of mitochondria’s involvement in cancer underscores the need for a comprehensive review synthesizing existing knowledge across diverse studies to identify critical knowledge gaps and inform future research directions [6].

This review aims to provide an in-depth analysis of mitochondria's role in human health and disease, with a particular focus on their involvement in cancer. To achieve this, we conducted a bibliometric analysis of scientific literature published over the past 30 years (1990–2023) to evaluate research trends, identify the most frequently studied cancer types linked to mitochondrial dysfunction, and determine the leading contributing countries in this field. Additionally, we focus on liver cancer, a global health challenge with complex metabolic underpinnings, to assess the current state of research and highlight potential avenues for therapeutic advancements. By integrating a quantitative bibliometric approach with a mechanistic review of mitochondrial function in cancer, this study provides a comprehensive perspective that can guide future research and therapeutic innovation.

## Mitochondria are crucial players in human health and disease

**Fig. 1:**
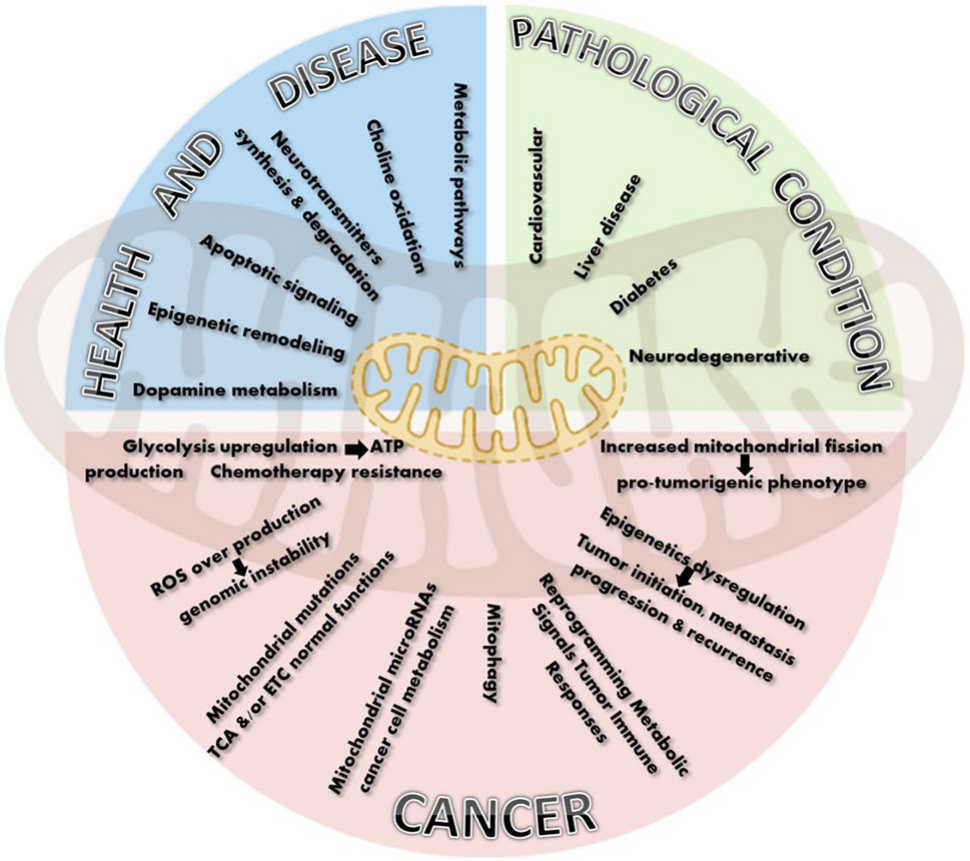
Schematic representation illustrating the diverse and intricate functions of mitochondria across various conditions, highlighting their essential role in human health and disease

Mitochondria play a crucial role in maintaining homeostasis and overall human health by performing a diverse range of complex functions (Fig. 1). Due to their unique structure, they are involved in major metabolic pathways including lysine metabolism [7], malate–aspartate shuttle system [8], protein acetylation [9] and lipid beta-oxidation of long-chain, medium-chain and short-chain fatty acids into acetyl-CoA [10]. In addition, mitochondrial choline oxidation leads to synthesis of structural precursors for lipoproteins, cardiolipin, phosphatidylethanolamine and membrane lipids [11, 12]. They play a vital role in apoptotic signalling where apoptosis is induced by BAK (Bcl2 antagonist/killer) and BAX (Bcl2 associated X-protein) causing OMM permeabilization, cytochrome c release and activation of apoptotic caspases [13].

Additionally, mitochondria are involved in epigenetic remodelling through the metabolic generation of citrate which is metabolized to acetyl-CoA used for histone acetylation [14, 15]. Neurotransmitters synthesis and degradation is another aspect in which mitochondria are involved. Mitochondria participate in dopamine metabolism besides the synthesis of catecholamines and neurotransmitters such as serotonin, melatonin, norepinephrine and epinephrine [16, 17]. Furthermore, they induce immunological reactions via activating inflammasome and interferon signalling [18]. Taken together, mitochondria are indispensable for normal physiological functions.

Mitochondrial dysfunction is linked to various diseases including cardiovascular, neurodegenerative, diabetes, and liver diseases, and most importantly cancer. In cardiovascular diseases such as ischemic heart disease, ultrastructural details of ischemic cardiomyocyte display the accumulation of lipid deposits and swollen mitochondria leading to metabolic alterations [19]. Hence, many mitochondrial components are promising drug targets for managing ischemic heart disease. Such targets include mitochondrial permeability transition pore, mitochondrial potassium channels and respiratory chain complexes inhibitors [20]. Additionally, mitochondria are linked to several neurodegenerative diseases such as Parkinson’s and Alzheimer’s disease. Parkinson’s disease develops due to defective nuclear transcriptional control of mitochondrial proteins leading to the accumulation of damaged mitochondria. The decline in the clearance of dysfunctional mitochondria through mitophagy results in Parkinson’s disease-induced neurodegeneration [21]. Moreover, impaired mitochondrial bioenergetic machinery adversely affects energy metabolism in Alzheimer’s disease. A large body of evidence demonstrates that reduced glucose utilization is one of the earliest and most consistent features of Alzheimer’s disease [22, 23]. Dysfunctional mitochondria are a major source of oxidative imbalance which is observed in Alzheimer’s disease. They become less efficient producers of ATP but more efficient producers of reactive oxygen species (ROS) [24].

Furthermore, disturbed calcium flux between the endoplasmic reticulum (ER) and the mitochondria due to dysfunctional proteins and channels involved in ER–mitochondrial Ca^2+^ transfer contributes to several liver diseases [25]. In type 2 diabetes abnormality in the mitochondria results in activation of ROS production and inflammation progression which in turn leads to disturbances in calcium balance and insulin signaling causing an imbalance in glucose homeostasis [26].

Mitophagy, or the selective degradation of mitochondria by autophagy, plays a major role in regulating metabolic homeostasis and type 2 diabetes development and it is well established that defective mitophagy is linked to the development of insulin resistance [27]. Therefore, mitophagy modulators can act as potential therapeutic agents for diabetes and neurodegenerative diseases [28]. Taken together, mitochondria are not only involved in energy production, but they are also central organelles whose dysfunction is linked to different diseases.

## Aberrant mitochondrial function in the development and progression of cancer

Mitochondria play an essential role in normal division of eukaryotic cells. Hence, dysfunctional mitochondria are crucial participants in cancer initiation and progression (Fig. 1). Cancer cells are known for reprogramming their metabolism where they become more glycolytic which is known as the Warburg effect. The latter refers to metabolizing glucose anaerobically via glycolysis rather than aerobically via OXPHOS even in the presence of functioning mitochondria under normoxia (normal oxygen levels) [29]. ATP production by upregulated glycolysis is not the primary objective of cancer cells. They utilize intermediates produced during glycolysis for macromolecules synthesis leading to tumor cells growth and proliferation. Such a metabolic shift in ATP production pathway also contributes to chemotherapy resistance [30]. Although not all cancer cells have dysfunctional mitochondria, important subsets do, possessing defective mitochondria with mutations in some proteins of TCA (tricarboxylic acid) cycle and subunits of electron transport chain (ETC) [31]. These mitochondrial mutations damage the normal function of TCA and/or ETC inducing tumor cells proliferation. Therefore, targeting mitochondrial metabolism is a promising strategy for cancer therapy [32].

Mitochondria in cancer cells are characterized by overproduction of ROS, which induces genomic instability and modified gene expression [33]. Even the metastatic potential of poorly metastatic cell lines can be enhanced by mtDNA (mitochondrial DNA) mutation of NADH (nicotinamide adenine dinucleotide + hydrogen) dehydrogenase subunit 6 (ND6). This mutation decreases the activity of respiratory complex I and is associated with increased ROS [34]. Additionally, a loss-of-function mutation of respiratory complex II, known as succinate dehydrogenase, leads to the accumulation of succinate in the mitochondria. The accumulated succinate then leaks into the cytoplasm impairing normal mitochondrial metabolism [35]. Besides mtDNA mutation, mitochondrial microRNAs (MitomiRs) are vital for cancer cells metabolism. MitomiRs are miRNAs of nuclear or mitochondrial origin that are localized in mitochondria. They have a crucial role in regulating mitochondrial oxidative capacity, ROS production and mitochondrial metabolism [36]. They control cancer cell metabolism by regulating mRNA expression and targeting key transporters or enzymes involved in cellular metabolism. Moreover, they can function as tumor suppressors that inhibit tumor cell proliferation. For example, miR-34a induces apoptosis through the downregulation of Bcl-2 (B-cell lymphoma/leukemia-2) and SIRT1 (silent information regulator sirtuin 1) [37]. Therefore, miR-34a is downregulated in breast cancer and its upregulation decreases cell proliferation and invasion. In addition, mitochondria influence tumor immune responses by reprogramming the metabolic signals in both cancer cells and immune cells residing in the tumor microenvironment [38, 39]. Under normal conditions, the transition of T-cells from naïve to effector T-cells is accompanied by cristae remodeling, leading to less efficient electron transfer and OXPHOS, which in turn induces glycolysis [40]. On the other hand, in immunosuppressive microenvironment T-cells are rendered dysfunctional. They demonstrate persistent loss of PPAR-γ (peroxisome proliferator-activated receptor gamma) coactivator 1α (PGC1α) which regulates mitochondrial biogenesis [41].

Macrophages are another immune cell which resides in tumor microenvironment. The polarization of macrophages to their pro-inflammatory phenotype is induced through the mitochondrial metabolic shift from OXPHOS to glycolysis [42]. On the contrary, tumor-associated macrophages (TAMs) undergo metabolic reprogramming towards reduced glycolysis. Hence, TAMs rely on OXPHOS, fatty acid oxidation and the upregulation of arginase 1 (ARG1). These metabolic changes trigger an immunosuppressive macrophage phenotype which induces pro-tumor signaling in the tumor microenvironment [43, 44].

Mitophagy plays an important role in tumorigenesis. It can act as a tumor promoter or suppressor depending on the type of cancer cells. It results in the elimination of defective mitochondria leading to reduced generation of ROS by functional mitochondria, which prevents carcinogenesis and limits the tumor initiating capacity of ROS [45]. Dysregulated mitophagy occurs due to impaired mitophagy receptors and adaptors including PINK1 (PTEN-induced kinase 1), Parkin and BNIP3 (Bcl2-interacting protein 3). Mitophagy-initiating proteins such as ULK1 (Unc-51-like autophagy activating kinase 1) play a significant role in breast cancer, where a decreased expression of ULK1 is associated with breast cancer progression [46]. ULK1 knockout mice demonstrate deficient mitophagy leading to breast cancer metastasis, especially to the bone, due to ROS-induced NLRP3 (nucleotide-binding oligomerization domain, leucine-rich repeat and pyrin domain-containing protein 3) inflammasome activation [47]. Conversely, once tumors are already in progress, mitophagy can act cytoprotectively by inducing tumor progression and suppressing chemotherapy-induced apoptosis. It can promote plasticity of cancer stem cells for better adaptation to the tumor microenvironment [45]. Controlling mitophagy can be achieved by modulating the transcription of mitophagy genes, controlling ROS production, and modulating major metabolic pathways such as the Krebs cycle or glutamine metabolism [48]. Hence, mitophagy is considered a potential target for cancer treatment.

Mitochondria and epigenetics play central roles in tumor initiation, progression metastasis and recurrence [49]. Mitochondria are important suppliers and regulators of multiple key metabolites that play pivotal roles in epigenetic regulation. These metabolites include acetyl-CoA, α-ketoglutarate (α-KG) and S-adenosyl methionine (SAM), which serve as the main substrates for DNA methylation and histone post-translational modifications [50]. Also, altered mitochondrial dynamics play an important role in cancer development, such as increased mitochondrial fission leading to a pro-tumorigenic phenotype [51].

Additionally, mitochondria can contribute to tumor angiogenesis via HIF-1α (hypoxia-inducible factor-1 alpha), which induces the expression of several angiogenic factors such as VEGF (vascular endothelial growth factor) and angiopoietin 2 [52]. They are also linked to multidrug resistance which occurs due to ATP-dependent multidrug efflux pumps that export chemotherapeutic agents out of the cell. Functional mitochondria assist multidrug resistance by providing sufficient ATP for ATP-dependent efflux pumps and stimulating Nrf2 (nuclear factor erythroid 2-related factor 2) function [53, 54].

Even the outcomes of radiation therapy are strongly connected to mitochondria which mediate radiation-induced signaling and cell death [55]. Collectively, mitochondria are key central players in various signaling networks that regulate tumorigenesis, progression and metastasis**.**

## Bibliometric and assessment approaches of scientific publications related to mitochondria over the period 1990–2023

### Bibliometric research methodology

Aiming to perform a bibliometric analysis on research linking mitochondria to cancer pathogenesis over the period 1990–2023, we extracted data from the PubMed database [56], which has a precise and specific search engine. After several iterations, we performed query strings and searched the PubMed database based not only on MeSH terms but also on the article title and a list of journals specialized in cancer, neoplasm, and tumors as well. The complete list of queries is provided in Supplementary Tables (Tables S1 and S2). Only articles and reviews were considered.

As PubMed does not allow analyses by country or institution, we used InCites which is an analytical tool developed and marketed by Clarivate Analytics [57]. This tool enables bibliometric analysis by country, discipline, and journal, etc., and provides bibliometric indicators. We also used the VOSviewer software developed by the Centre for Science and Technology Studies (Bibliometric Department of Leiden University, Leiden, Netherlands) [58], which enables us to graphically represent links between countries, authors or keywords in cluster form. For example, the closer two keywords are to each other, the more often they have been associated. Conversely, if two keywords are in distant clusters, then they are very rarely associated. The size of each colored circle is proportional to the total number of articles. Colors are used by the software in order to distinguish between the different clusters.

The impact of the research was assessed by analyzing the frequency of citations, which partly depends in part on the year of publication and the scientific field. As older articles have had more opportunity to be cited, it is essential to normalize citations by publication year and disciplinary field [59]. In Web of Science (WoS), journals are classified into 254 fields called WoS Categories. We used three classical bibliometric impact indicators: the number (and percentage) of articles classified as being either in the top 1% or in the top 10% (i.e. either the 1% or the 10% most cited articles globally, adjusted by publication year and WoS category). The Category Normalized Citation Impact (CNCI) is the ratio of the observed number of citations to an expected number of citations. A CNCI of two means that the publication was cited twice as often as the world average.

Finally, we used iCite [60], a tool developed by the NIH (USA) to access a dashboard of bibliometrics for papers associated with a portfolio. iCite has three modules: Influence, Translation, and Open Citations. For each publication, the Translation module analyzes the MeSH terms and calculates the percentage of terms relating to human beings (Human), animals (Animal) or molecular or cellular biology (Mol/Cell). This module can therefore be used to measure whether the research carried out is still basic, translational or clinical [61].

### Quantitative analysis of research relating mitochondria to cancer pathogenesis

**Fig. 2:**
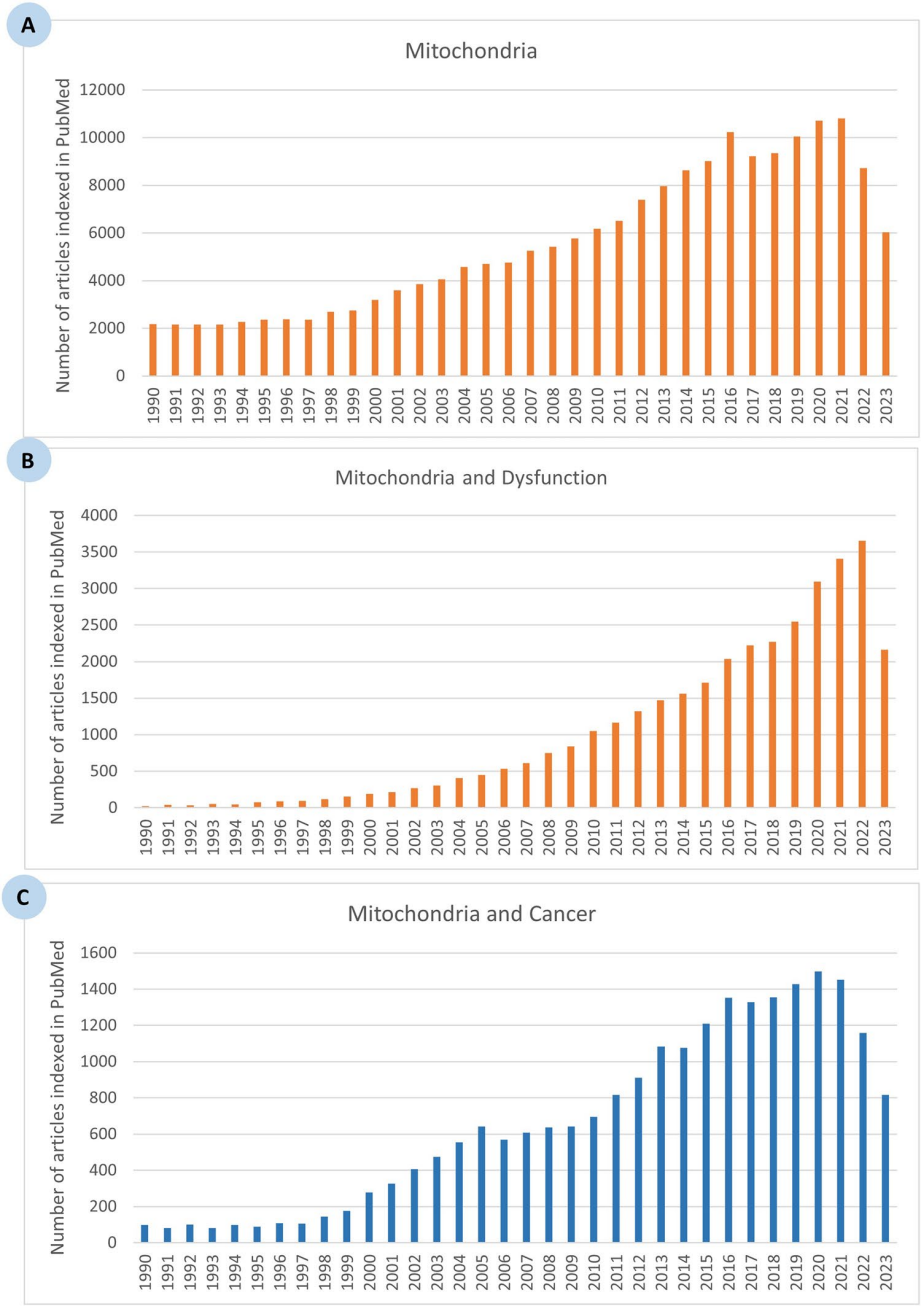
Bibliometric analysis of research articles published between 1990 and 2023, illustrating the number of articles indexed in PubMed **A** using only the Medical Subject Heading (MeSH) term ‘mitochondria’, **B** combining the search term ‘mitochondria’ and ‘dysfunction’, and **C** combining the search terms ‘mitochondria’ and ‘cancer’

Our bibliometric search in PubMed yielded 169,555 publications with the medical subject headings (MeSH) terms ‘mitochondria’ (Fig. 2A), of which 34,949 (20.61%) included the terms ‘mitochondria’ and ‘dysfunction’ (Fig. 2B) and 22,406 (13.21%) included the terms ‘mitochondria’ and ‘cancer’ (Fig. 2C). It should be noted from these data that not all mitochondrial dysfunctions lead to cancer or enhance its progression, which is in accordance with previous studies [39, 62]. Moreover, our investigation revealed that the research output relating mitochondria to cancer has expanded markedly over time, with a four-fold increase in the number of articles indexed in PubMed from 1366 to 5555 articles over the period 1990–2000 and 2000–2010, respectively. Over the last decade, our analysis indicated a 178.75% increase in the number of articles indexed in PubMed, reaching 15,485 articles over the period 2011–2023 instead of only 5555 articles over the period 2000–2010. This expansion represents an overall 11-fold increase in the number of publications linking mitochondria to cancer since 1990, whereas only a three-fold increase has been recorded since 2011.

This substantial increase in the number of articles linking mitochondria to cancer since 1990 demonstrates the growing scientific interest in this field. However, the slower growth rate over the last decade may suggest that the field is approaching a phase of maturity, where foundational discoveries have been established, and researchers are now focusing on more specific aspects of mitochondrial dysfunction in cancer. This shift may indicate a transition from broad exploratory studies to more targeted investigations aimed at understanding the precise mechanisms by which mitochondrial alterations contribute to tumorigenesis and identifying potential therapeutic strategies [35].

**Fig. 3:**
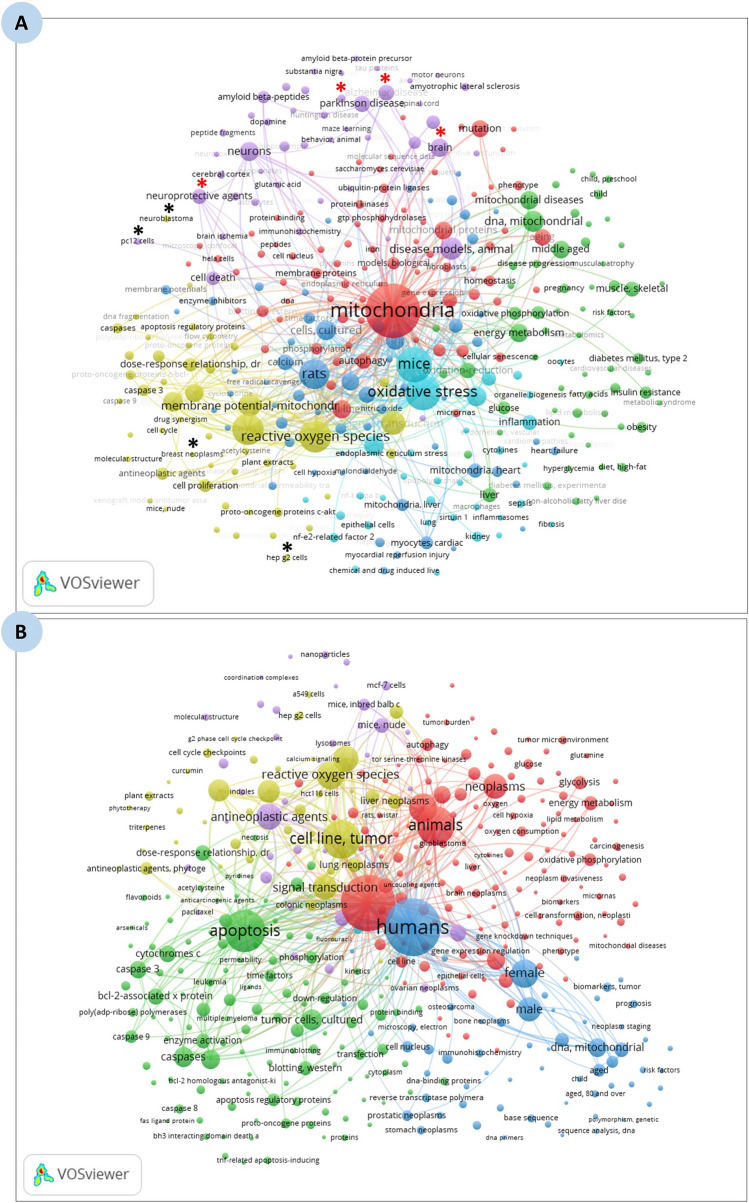
VOSviewer-generated schematic representation illustrating **A** the majority of extracted publications focus on neurodegenerative diseases, particularly Parkinson’s and Alzheimer (*red asterisk*), with comparatively fewer studies on cancer (*black asterisk*), and **B** the numbers of articles linking mitochondria to various types of cancer/neoplasms. The size of each colored circle corresponds to the total number of articles, while different colors differentiate distinct research clusters

Figure 3A visualizes the interplay between mitochondria and various diseases and pathologies. This diagram demonstrated that most of the articles were related to neurodegenerative diseases, particularly Parkinson’s and Alzheimer’s, while only a few focused on cancer. This aligns with the fundamental role of mitochondria in cellular energy metabolism and neuronal health, as neurons are highly dependent on mitochondrial function [21–24]. The lower proportion of mitochondrial research focusing on cancer could indicate that while mitochondria play a role in tumorigenesis, other pathways (e.g., genetic mutations, immune evasion, and metabolic reprogramming) are more prominently studied in cancer biology. Second, to deepen our literature search, several specific keywords related to mitochondria and/or cancer and/or neoplasm covering all types of cancer were used. We considered only the data from MeSH terms with at least 100 articles. Our analysis showed that cancer and/or neoplasm in the liver (2569 articles) ranked first, followed by the brain (1840 articles), the lung (1548 articles), and finally the breast (1469 articles). In addition, various organs, including but not limited to the colorectum, colon, prostate, stomach, blood, and pancreas, showed a lower number of published articles (n < 700). Other organs like the oesophagus, thyroid, head & neck, and urinary bladder exhibited a significant decrease in the number of articles (n < 150). The observed differences in the number of publications related to cancer and/or neoplasms may reflect the higher prevalence of certain cancer types compared to others. These findings were graphically represented by the VOSviewer software (Fig. 3B). The prominence of liver, brain, lung, and breast cancers in mitochondrial research may be influenced by variations in disease prevalence, severity, or the distinct role of mitochondria in each cancer type [32]. Liver cancer’s leading position in this field could stem from its strong association with metabolic disorders, hepatitis infections, and environmental toxins, all of which directly affect mitochondrial function [63]. In contrast, cancers such as those of the thyroid, bladder, and head & neck, which have fewer related publications, may be less dependent on mitochondrial dysfunction as a key factor in tumor development.

Between 1990 and 2023, 169,555 articles about mitochondria were found in PubMed versus 158,353 articles (93.39%) in WoS after transfer, which were also accessible via the InCites platform. Subsequently, we analyzed the disciplines of the journals in which these publications were published. In WoS and InCites, the disciplines of the journals were classified into 166 categories (Table 1). Overall, neuroscience was strongly represented (9.84%), whereas cancer-related research accounted for only 4.7% of publications, supporting our previous finding that not all mitochondrial dysfunctions lead to cancer or enhance its progression. Focusing on cancer-related articles, we found that 1.09% of publications were in the top 1% most cited articles, while 14.02% were in the top 10% most cited articles. This citation analysis shows that despite being a smaller subset, mitochondrial research in cancer is highly influential, with 14.02% of these articles ranking in the top 10% most cited publications, potentially driving new therapeutic approaches [35].

**Table 1:** Referring to the query mitochondria: the top thirty disciplines of the journals, category normalized citation impacts, and the number of articles in the top 1% as well as in the top 10% journals over the period 1980 to 2023

Disciplines of journals	Web of science documents	Category normalized citation impact	Documents in top 1%	Documents in top 10%
*Total*	158,353	1.34	2864	26,412
Biochemistry & Molecular Biology	51,606	1.32	811	7588
Cell Biology	29,551	1.27	434	4157
Genetics & Heredity	20,067	0.89	106	1816
Neurosciences	11,538	1.33	165	1694
Pharmacology & Pharmacy	10,334	1.42	125	1676
Biophysics	9525	1.20	119	1201
Endocrinology & Metabolism	8356	1.71	221	1770
Oncology	7448	1.21	81	1044
Medicine, Research & Experimental	7371	1.54	161	1266
Physiology	6704	1.46	144	1207
Toxicology	5439	1.37	76	863
Chemistry, Multidisciplinary	4081	1.07	34	371
Evolutionary Biology	4042	1.19	31	510
Clinical Neurology	3996	1.39	64	611
Cardiac & Cardiovascular Systems	3922	1.86	68	941
Biology	3745	1.59	82	687
Plant Sciences	3466	1.52	49	632
Biotechnology & Applied Microbiology	3078	1.03	31	301
Immunology	2826	1.54	82	463
Biochemical Research Methods	2777	1.05	31	315
Chemistry, Medicinal	2717	1.29	31	379
Microbiology	2455	0.88	7	187
Geriatrics & Gerontology	2219	1.57	46	437
Peripheral Vascular Disease	1853	1.89	51	483
Hematology	1718	1.94	49	436
Food Science & Technology	1705	1.29	8	227
Pathology	1667	1.37	22	261
Nutrition & Dietetics	1627	1.07	11	157
Developmental Biology	1617	1.44	42	265
Reproductive Biology	1565	1.35	21	263

### Evolution of contributions from major countries and analysis of the eight 4-year intervals within the study period

**Fig. 4:**
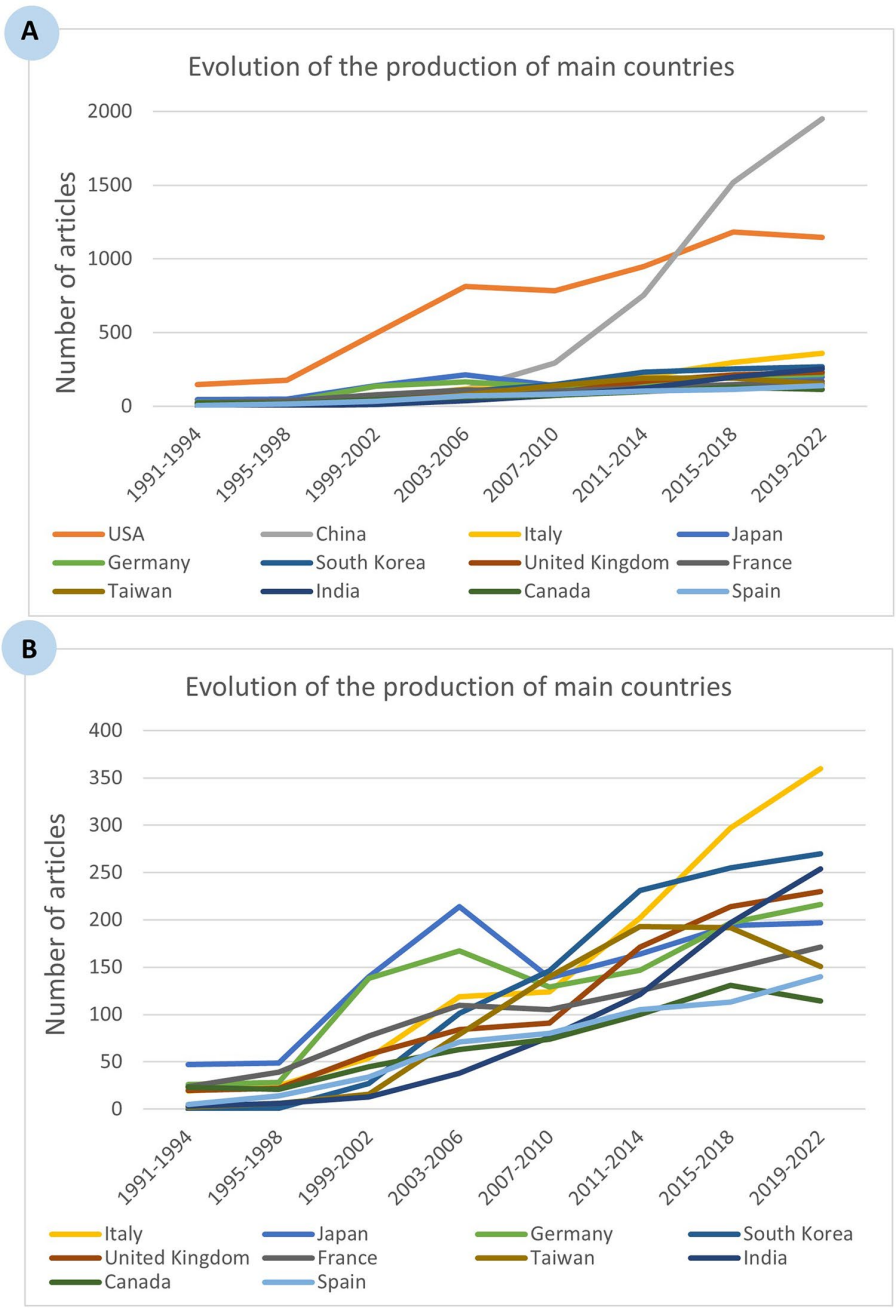
Trend analysis of research contributions on mitochondria in cancer from major countries across eight 4-year intervals between 1991 and 2022, highlighting **A** the USA and China as the leading contributors in publication volume, and **B** the distribution of contributions from other countries, excluding the USA and China, for clearer data visualization

From 1991 to 2022, after transfer from Pubmed, we found 18,333 articles on mitochondria and cancer in WoS and InCites. We looked at the contributions from major countries to the global output relating to mitochondria and cancer (Fig. 4). Table 2 shows the top twenty countries over the period 1991–2022 with the highest number of publications. The USA occupied the first position among countries (5695 articles), followed by China (4660 articles), Italy (1200 articles), and Japan (1144 articles). Egypt came in the thirty-eighth position with 84 publications representing 0.46%. It is worth noting that local publications in Egypt do not appear via this analysis leading to a reduced percentage that does not mimic the real size of scientific research in Egypt.

**Table 2:** Referring to the query mitochondria and cancer: overall numbers of articles and numbers that are in the top 1 and 10% most cited articles by the top twenty countries over the period 1991–2022

Countries	Web of science documents	Category normalized citation impact	Documents in top 1%	Documents in top 10%
*World*	18,333	1.39	335	3334
USA	5695	1.84	207	1459
China	4660	1.32	56	816
Italy	1200	1.38	18	243
Japan	1144	1.08	10	140
Germany (Fed Rep Ger)	1048	1.47	26	195
South Korea	1031	1.13	5	146
United Kingdom	890	1.89	30	255
France	799	1.67	24	197
Taiwan	779	1.20	5	107
India	708	1.17	11	102
Canada	571	1.52	11	121
Spain	562	1.55	12	123
Australia	386	1.61	10	88
Sweden	298	1.36	5	55
Poland	284	1.01	1	32
Brazil	256	1.22	3	37
Switzerland	226	2.04	10	63
Netherlands	217	1.66	7	46
Czech Republic	193	1.13	3	29
Hong Kong	180	1.39	2	36

Importantly, the USA is the first-ranked country having both the top 1% and 10% impact indicators with 207 and 1459 articles, respectively. China is still positioned second with 56 and 816 articles corresponding to the top 1 and 10% most cited articles, respectively. Briefly, the USA and China are the leaders in this field.

Worldwide, the percentage increase in the number of publications related to mitochondria and cancer, as shown in Table 3, is much higher over the period 1991–2022 (1,347.48) than in the last decade (55.05). Table 3A presents the number of articles published by the top five countries across the eight 4-year intervals over the period 1990 to 2023. Among these countries, China showed the highest percentage increase (64,900), followed by Italy (1,794.74), and Germany (730.77) within the overall analysis period.

**Table 3:** Referring to the query mitochondria and cancer: Overall numbers of articles by the top five countries (A) over the period 1991–2022, and (B) within the last decade

	Total	(A) Period 1991–2022			(B) Last decade 2011–2022		
		Number of articles			Number of articles		
		1991–1994	2019–2022	Change (%)	2011–2014	2019–2022	Change (%)
*Worldwide*	18,333	337	4878	1347.48	3146	4878	55.05
USA	5695	148	1147	675	948	1147	20.99
China	4660	3	1950	64,900	754	1950	158.62
Italy	1200	19	360	1794.74	202	360	78.22
Japan	1144	47	197	319.15	164	197	20.12
Germany	1048	26	216	730.77	147	216	46.94

Even though the USA occupied the first position regarding the number of publications as well as the top 1 and 10% impact indicators among countries, it drops to the fourth position with respect to its percentage change (675). This finding indicates that research output growth tends to be stable in the USA over the study period with a more solid research foundation and better quality results in this field. Meanwhile, China showed a rapid increase in output due to its substantial support for scientific research over the last couple of decades. Japan showed the lowest percentage increase (319.15%) in the number of publications within the overall analysis period. Focusing on the last decade, the top five countries maintain the same order (Table 3B), in terms of percentage change, with China in the first position (158.62), followed by Italy (78.22), Germany (46.94), the USA (20.99), and Japan (20.12).

These findings highlight key trends in global research output on mitochondria and cancer. The substantial increase in China’s research output reflects significant national investment in scientific research. Meanwhile, Italy and Germany have also demonstrated impressive growth, whereas Japan has shown more modest expansion. The USA's stable research productivity suggests a mature phase with a strong foundation, while China's rapid rise indicates ongoing expansion. Egypt’s limited representation (0.46%) may stem from the exclusion of local journals from major databases. These trends highlight disparities in funding, scientific priorities, and infrastructure, emphasizing the need for greater international collaboration and innovation in mitochondrial cancer research.

### Evolution of the top four cancer types and analysis of the eight 4-year intervals within the study period

Therefore, we focused our interest on analyzing the most frequent type of cancer/neoplasm worldwide within the study period. The bibliometric search in PubMed yielded 169,555 publications with the MeSH term ‘mitochondria’. Meanwhile, the search identified 332,391, 253,469, 205,324, and 155,678 publications for ‘breast neoplasms’, ‘lung neoplasms’, ‘brain neoplasms’, and ‘liver neoplasms’, respectively (Table 4). However, cross-referencing the term ‘mitochondria’ with any of these terms returned only 1701 (0.51%) for ‘breast neoplasms’, 1165 (0.46%) for ‘lung neoplasms’, 1792 (0.87%) for ‘brain neoplasms’, and 1336 (0.86%) for ‘liver neoplasms’. These findings demonstrate that mitochondrial aberrations are more pronounced in the brain (0.87%) than in the liver (0.86%), the breast (0.51%), or the lung (0,46%).

**Table 4:** Overall numbers of articles by the top four cancer types with or without the MeSH term “mitochondria” showing the percentage change either within the overall period 1991 – 2022 or within the last decade

	Total		Number of articles			Change (%)	
	Without MeSH term ‘mitochondria’	Two MeSH terms ‘mitochondria’ and ‘neoplasms’	1991–1994	2011–2014	2019–2022	Period 1991–2022	Last decade
						1991–2022	2011–2022
*Organ*	–	165,555	–	–	–	–	–
Brain	205,324	1792	29	355	445	1434.48	25.35
Liver	155,678	1336	70	258	360	414.29	39.53
Breast	332,391	1701	11	376	515	4581.82	36.97
Lung	253,469	1165	12	270	340	2733.33	25.93

**Fig. 5:**
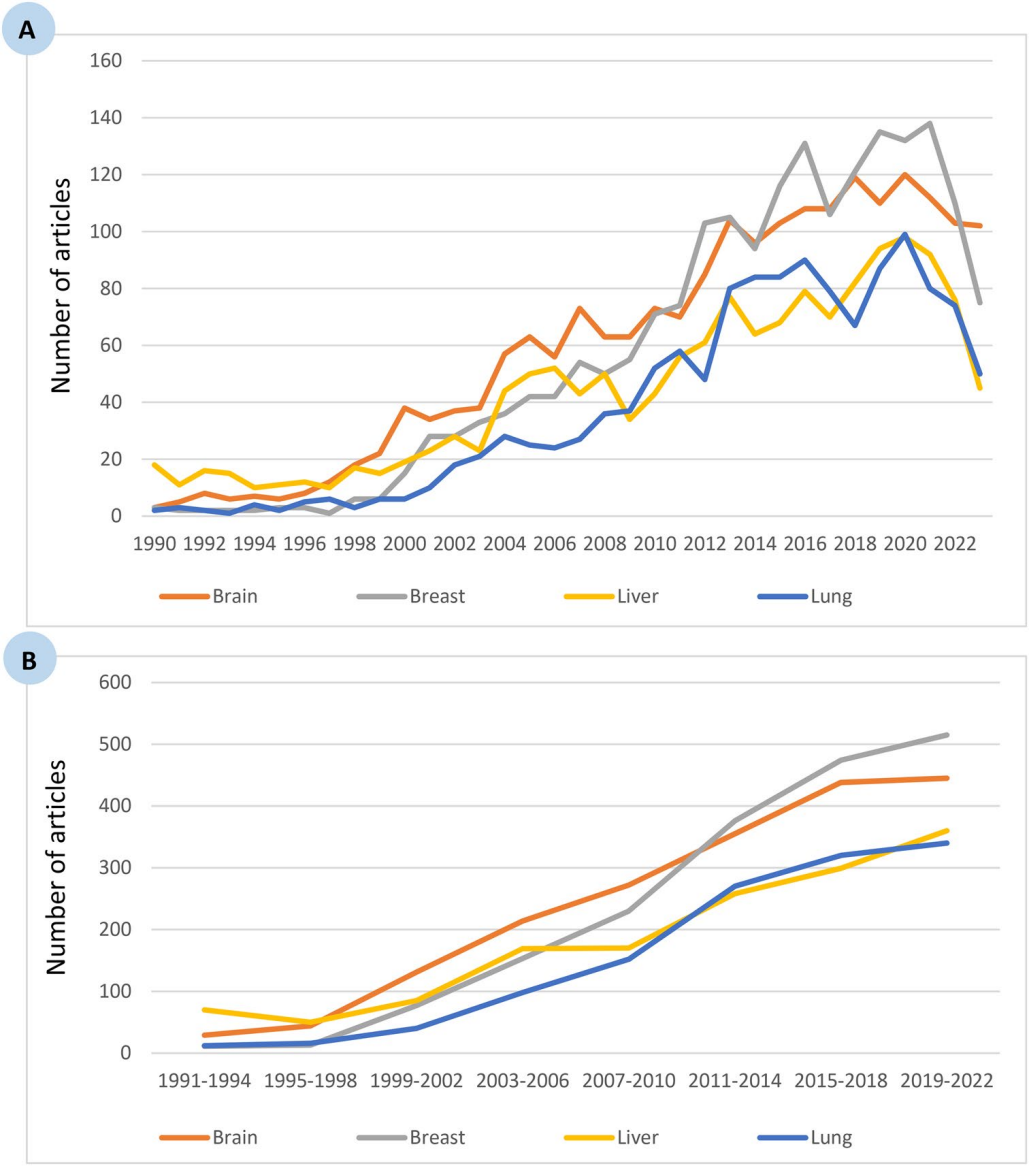
Analysis of the publication trends on different types of cancer/neoplasms, illustrating **A** the overall growth in research output from 1990 to 2023, and **B** the distribution of publications across eight 4-year intervals within the study period

Regarding the different types of cancer/neoplasm, the evolution of the production of articles has continuously increased in numbers throughout the study period from 1990 to 2020, but a slight decrease has been reported since 2020, possibly due to the COVID-19 pandemic lockdown across the globe (Fig. 5A).

In the next step of our literature search, we focused on the analysis of published articles on each type of cancer/neoplasm within the eight 4-year intervals of the study period (Fig. 5B; Table 5). Our results revealed that the percentage change was highest for breast neoplasms (4581.82), followed by lung neoplasms (2733.33), brain neoplasms (1434.48), and liver neoplasms (414.29) over the study period from 1990 to 2023. However, focusing on the last decade, our data showed the highest percentage increase for liver neoplasms (39.53), followed by breast neoplasms (36.97), lung neoplasms (25.93), and brain neoplasms (25.35). This trend may be attributed to the fact that the incidence rate of brain cancer/neoplasms has remained relatively stable from 2011 to 2022, whereas liver cancer/neoplasms have exhibited a continuous increase each year in the last decade (Table 5). Moreover, liver cancer is particularly prevalent in Egypt, further highlighting its global significance. Given these observations, we have chosen to focus on liver cancer/neoplasms in the next step of our bibliometric analysis to better understand its association with mitochondrial dysfunction and its growing impact on global health.

**Table 5:** Overall numbers of articles by the different cancer types showing the percentage change either within the overall period 1991–2022 or within the last decade

	(A) Period 1991–2022			(B) Last decade 2011–2022		
	Number of articles			Number of articles		
	1991–1994	2019–2022	Change (%)	2011–2014	2019–2022	Change (%)
*Organ*						
Brain	29	445	1434.48	355	445	25.35
Liver	70	360	414.29	258	360	39.53
Breast	11	515	4581.82	376	515	36.97
Lung	12	340	2733.33	270	340	25.93

### Analysis of the liver cancer/neoplasms worldwide over the study period

Liver cancer is a pressing global health challenge that will lead to an estimated incidence of more than one million cases by 2025 [64]. It ranks as the sixth most common cancer and the third leading cause of cancer-related deaths worldwide [65]. Liver cancer includes hepatocellular carcinoma, which is responsible for 75–85% of cases, and intrahepatic cholangiocarcinoma, which accounts for 10–15% of cases [6]. It is one of the tumors with the lowest five-year survival rate after pancreatic cancer [66].

Liver cancer is attributed to different predisposing factors such as obesity, non-alcoholic fatty liver disease (NAFLD), type II diabetes, hepatitis B virus (HBV) and hepatitis C virus (HCV) [67]. According to health registries, different countries show different rates of liver cancer cases and deaths. In the USA, it has been reported that liver cancer will account for 41,210 new cases and 29,380 total deaths by the end of 2023 [66]. Japan is another industrial country with a high liver cancer incidence rate. According to the Japan Society of Hepatology, the annual death rate from liver cancer is 32,000 and is expected to rise over the next ten years due to the high prevalence of HCV [68]. Moreover, China is considered one of the main countries suffering from high burden of liver cancer due to the high prevalence of HBV. It accounts for more than 50% of the world's burden with approximately 2,814,000 patient deaths from the disease each year [69, 70]. In Egypt, liver cancer represents the fourth most common type of cancer [71, 72]. Although HBV is a major risk factor for liver cancer development, HCV contributes to the high prevalence of liver cancer in Egypt [73].

**Fig. 6:**
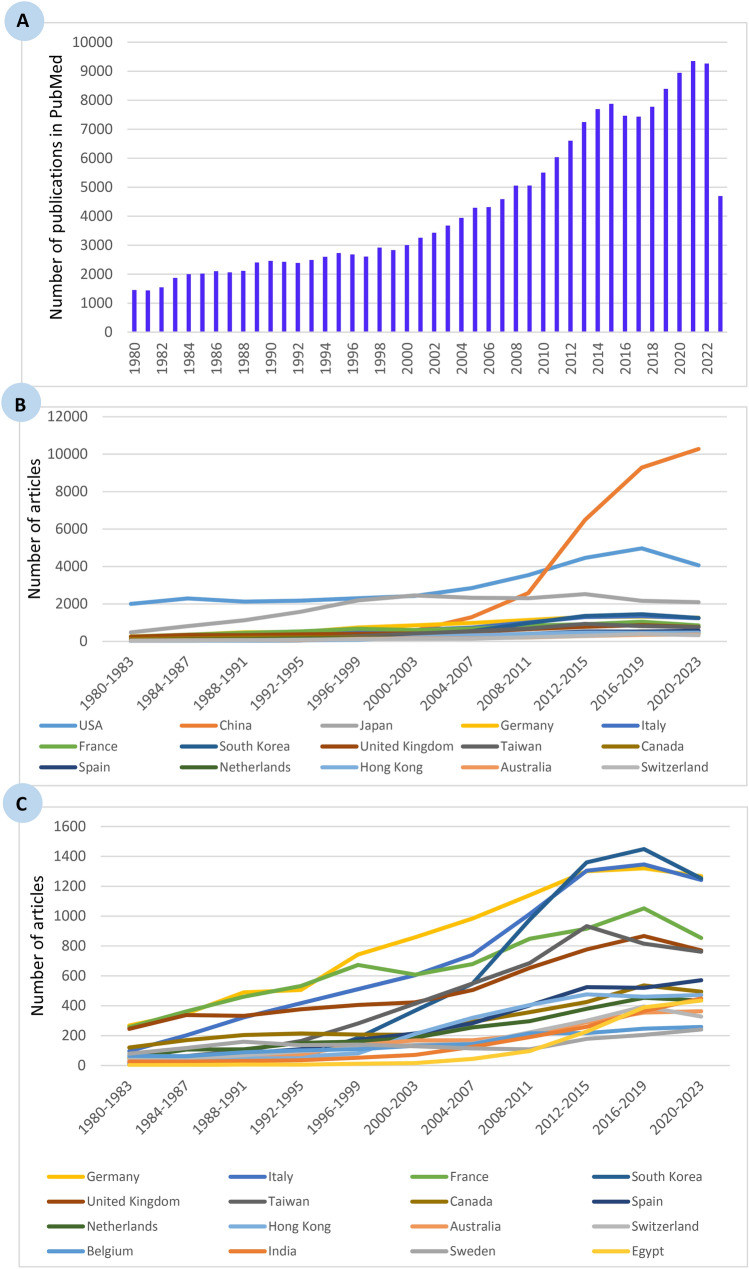
Bibliometric analysis of research articles published from 1980 to 2023, illustrating **A** the number of articles indexed in PubMed using the MeSH terms ‘liver neoplasms,’ ‘hepatocellular carcinoma,’ or ‘hepatic cancer,’ along with the evolution of contributions from major countries across eleven 4-year intervals, **B** research output including the USA and China, the two leading contributors, and **C** research output excluding the USA and China to enhance data clarity

Our bibliometric search supported these data and yielded 193,404 publications using the MeSH terms ‘liver neoplasms’, ‘hepatocellular carcinoma’, or ‘hepatic cancer’. After transfer, 173,620 publications were indexed in WoS, and they were also accessible via the InCites platform over the period 1980 to 2023 (Fig. 6A).

Regarding the contributions from major countries to liver cancer, our analysis once again supported the published data and highlighted the USA, China and Japan as the top three countries with the highest number of publications on liver cancer (Fig. 6B, C). Table 6 lists the top twenty-five countries across the eleven four-year intervals over the period 1980–2023, ranked by the highest number of publications. The USA produced the highest number of publications in liver cancer and occupied the first position from the 1980–1983 interval until the last decade, when its position shifted to the second rank, surpassed by China, which became the top-ranked country in terms of the overall number of publications from the 2012–2015 to 2020–2023 intervals. Japan maintained its third position from the beginning to the present. Meanwhile, Egypt ranked nineteenth with 435 publications in the 2020–2023 interval, compared to 227 publications in the 2012–2015 interval, representing a 91.63% increase over the last decade. It is particularly interesting to note that Egypt’s total research and development fund is very limited and scientists tend to publish their work in local journals instead of international ones indexed in PubMed and WoS.

**Table 6:** Referring to the query liver cancer/neoplasms: Overall numbers of articles by the top twenty-five countries in the eleven four-year intervals over the period 1980–2023

Country	1980–1983	1984–1987	1988–1991	1992–1995	1996–1999	2000–2003	2004–2007	2008–2011	2012–2015	2016–2019	2020–2023
*World*	4179	5405	5975	6627	8273	9570	11,519	14,597	20,480	22,869	22,379
USA	2004	2297	2122	2173	2306	2427	2847	3536	4460	4970	4062
China	23	49	80	105	181	571	1289	2582	6492	9290	10,277
Japan	482	811	1131	1586	2192	2452	2329	2308	2522	2164	2094
Germany	268	348	489	506	743	858	984	1140	1301	1320	1268
Italy	96	202	322	417	511	605	740	1014	1304	1346	1242
France	257	362	460	532	673	608	679	848	916	1052	854
South Korea	3	10	27	51	185	368	548	976	1360	1449	1253
United Kingdom	245	338	332	376	405	423	505	653	777	867	770
Taiwan	12	63	99	163	281	414	549	685	933	815	762
Canada	120	170	204	214	207	206	292	356	425	536	494
Spain	27	29	82	109	172	215	283	402	524	520	571
Netherlands	49	107	108	151	160	186	255	296	380	453	435
Hong Kong	19	23	48	60	81	211	319	405	476	461	472
Australia	46	64	74	75	136	168	169	206	289	354	363
Switzerland	40	47	69	95	116	138	146	221	300	395	328
Belgium	64	62	88	101	109	132	143	215	217	246	258
India	27	26	30	35	52	71	125	190	259	365	449
Sweden	79	117	159	136	140	129	114	108	178	205	241
Egypt	2	3	6	5	12	16	44	97	227	389	435
Austria	37	46	45	53	80	113	93	156	184	197	188
Singapore	8	4	10	14	28	67	87	181	220	247	307
Turkey	0	2	7	5	33	66	117	154	210	206	325
Greece	13	13	13	34	53	84	107	144	146	176	133
Brazil	2	6	6	10	17	32	65	127	174	210	218
Poland	26	13	31	36	44	44	64	82	108	130	142

Therefore, it would be important to conduct an in-depth analysis, if possible, of local research on liver cancer in Egypt, as it is the fourth most frequent type of cancer. Recently, Egypt, represented by the Science, Technology & Innovation Funding Authority (STDF) in cooperation with the Egyptian Knowledge Bank (EKB), signed a transformative Plus fully open-access agreement with Springer Nature (https://www.springernature.com/gp/open-research/oa-agreements/egypt), allowing Egyptian scientists to publish their work free of charge. This explains the recent considerable increase in scientific production from Egypt.

**Fig. 7:**
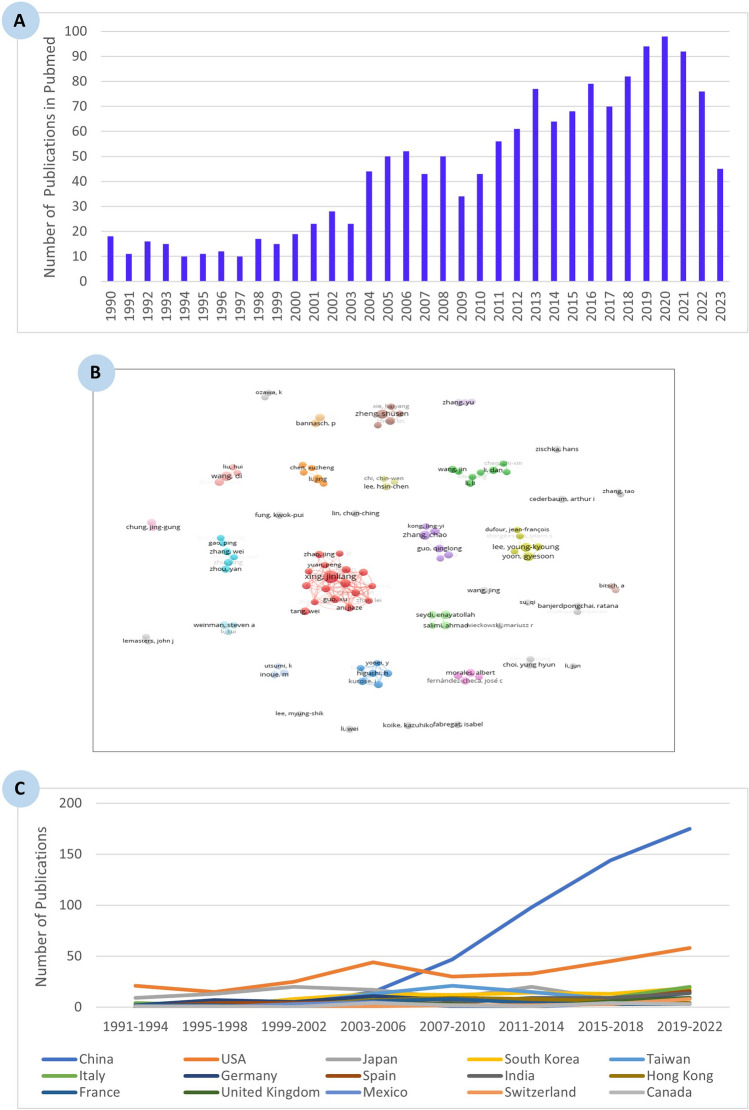
Bibliometric analysis of research articles published from 1990 to 2023, presenting **A** the number of articles indexed in PubMed using the MeSH terms ‘liver neoplasms’ and ‘mitochondria,’ **B** leading global authors with the highest number of publications on mitochondria in liver neoplasms, and **C** the evolution of research contributions from major countries across eight 4-year intervals within the study period

Based on the retrieved documents, we carried out a specific query by crossing the query ‘liver neoplasms’ (155,678) with the query ‘mitochondria’ (169,555) and identified 1336 (1231 after transfer to WoS) articles in PubMed over the study period 1990–2023 (Fig. 7A). Among these publications, research areas were classified into 65 categories (Table S3) with the highest percentage of documents classified under biochemistry and molecular biology (28.92%), followed by oncology (23.31%), cell biology (17.95%), and pharmacology and pharmacy (14.30%) categories.

Meanwhile, the highest number of articles in the top 10% were related to oncology (45 out of 215, 20.93%), followed by biochemistry and molecular biology (40 out of 215, 18.60%), and pharmacology and pharmacy (38 out of 215, 17.67%) categories. However, the highest number of articles in the top 1% were in different categories, namely gastroenterology and hepatology (28.57%) followed by toxicology (14.29%).

An analysis by journal was also performed and yielded 412 journals. The 1231 articles have been cited approximately 50,836 times during the study period 1990–2023. Table 7 lists the journals with at least a mean of 9 citations per article. The most cited articles were published in *Cancer Research* (31 documents cited 3506 times), followed by *Journal of Biological Chemistry* (34 documents cited 3058 times), *Cancer Letters* (29 documents cited 1466 times), *Proceedings of the National Academy of Sciences (PNAS) of The United States of America* (9 documents cited 1402 times), and *Free Radical Biology and Medicine* (25 documents cited 1374 times).

**Table 7:** Referring to the query mitochondria crossed with the query liver cancer/neoplasms: A list of the journals having at least a mean of 9 citations per article over the period 1980—2023

Name of journal	Web of science documents	Times cited	Category normalized citation impact
*Baseline for all Items*	1231	50,836	1.32
Biochemical and Biophysical Research Communications	35	991	0.93
Journal of Biological Chemistry	34	3058	1.65
Cancer Research	31	3506	2.38
Cancer Letters	29	1466	1.30
International Journal of Molecular Sciences	27	267	0.86
Free Radical Biology and Medicine	25	1374	1.42
Oncology Reports	22	372	0.63
Hepatology	21	944	1.53
Cell Death & Disease	21	648	1.30
Plos One	17	546	1.10
World Journal of Gastroenterology	16	561	1.01
International Journal of Oncology	16	430	0.72
Molecular Medicine Reports	16	238	1.30
Scientific Reports	15	398	0.98
Biochemical Pharmacology	15	416	1.40
Oncogene	14	1243	2.20
Biomedicine & Pharmacotherapy	14	206	0.99
Febs Letters	12	493	0.86
European Journal of Pharmacology	12	392	1.33
Chemico-Biological Interactions	11	292	1.14
Toxicology and Applied Pharmacology	11	879	2.22
Molecules	10	256	0.50
Carcinogenesis	10	514	1.37
Archives of Biochemistry and Biophysics	10	280	0.87
Proceedings of the National Academy of Sciences (PNAS) of The United States of America	9	1402	3.00
Journal of Ethnopharmacology	9	274	1.61
Food and Chemical Toxicology	9	378	1.68
Journal of Hepatology	9	951	4.79

Regarding the authors of these 1231 publications, they were from China, Korea, Iran, Spain, Germany, Taiwan, the USA, and other countries (Fig. 7B). The size of each colored circle is proportional to the total number of articles by that author. It should be noted that there are a very large number of unconnected clusters, reflecting a lack of collaboration and coordination at the global level. This finding indicates potential opportunities for increased international cooperation to advance this field. The output from China remained predominant over this period. The top ten authors with the highest number of publications are provided in Table 8.

**Table 8:** Referring to the query mitochondria crossed with the query liver cancer/neoplasms: a list of the top ten authors having the biggest number of publications related to mitochondria in liver neoplasms over the period 1990–2023

Authors	Number of documents
Xing, Jinliang	16
Wang, Di	9
Lee, Young-kyoung	8
Yoon, Gyesoon	8
Zhang, Chao	8
Zheng, Shusen	8
Huang, Qichao	7
Li, Jing	7
Seydi, Enayatollah	7
An, Jiaze	6

Worldwide, the percentage increase in the number of publications related to mitochondria and liver neoplasms, as shown in Table 9, was much higher over the period 1991–2022 (555.10) than in the last decade (54.33). Table 9A presents the top ten countries with the highest number of articles published in the eight four-year intervals over the period 1990 to 2023. Among countries, Spain showed the highest percentage increase (1500%), followed by Germany (600%), Italy (400%), the USA (176.19%), and Japan (66.66%) within the overall analysis period.

**Table 9:** The top ten countries having the highest number of articles related to mitochondria in liver neoplasms (A) over the period 1991 – 2022, and (B) within the last decade

	(A) Period 1991–2022			(B) Last decade 2011–2022		
	Number of articles			Number of articles		
	1991–1994	2019–2022	Change (%)	2011–2014	2019–2022	Change (%)
*Worldwide*	49	321	555.10	208	321	54.33
China	0	175	–	98	175	78.57
USA	21	58	176.19	33	58	75.75
Japan	9	15	66.66	20	15	−25
South Korea	0	19	–	14	19	35.71
Taiwan	0	9	–	15	9	−40
Italy	4	20	400	7	20	185.71
Germany	2	14	600	7	14	100
Spain	1	16	1500	7	16	128.57
India	0	14	–	9	14	55.55
Hong Kong	0	4	–	8	4	-50

Focusing on the last decade, Italy displayed the highest percentage increase (185.71%) regarding the number of publications among countries, whereas Spain dropped to the second position in terms of percentage change (128.57%). Several countries including Japan, Taiwan, and Hong Kong, experienced a percentage decrease in the number of publications over the last decade of 25, 40, and 50%, respectively (Table 9B).

**Fig. 8:**
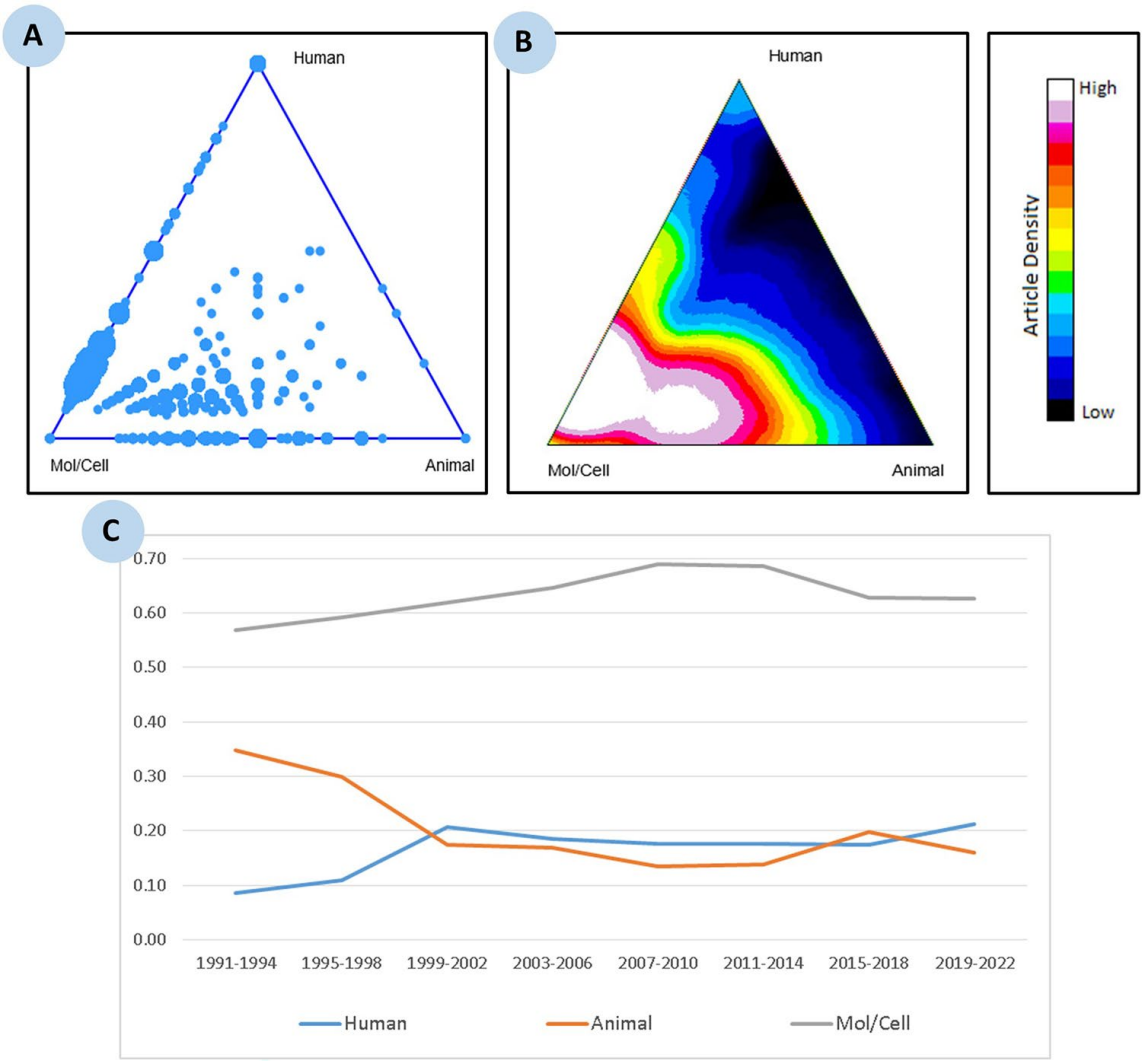
Research on mitochondria in liver cancer: **A**, **B** distribution of publications based on iCite’s Translation module, and **C** trends in publication output across eight 4-year intervals

Finally, an analysis of publications using iCites' Translation module shows that research is still very much focused on cellular and molecular biology (Fig. 8A, B). Figure 8C shows that molecular and cellular biology accounts for 60% of publications, animal research for 20% and human research for 20%. These percentages have changed little over the last 30 years. These results are consistent with the analysis of WoS categories and journals as well as with the implementation and application of the 3Rs principle in animal experimentation [74, 75].

To sum up, a key limitation of this study is the lack of analysis of local research that is not indexed in major international databases such as PubMed and Web of Science. In many countries, particularly those with limited research funding, scientists often publish their work in national or regional journals that may not be widely accessible. As a result, valuable contributions to cancer research may be underrepresented in our analysis. However, recent initiatives promoting open-access publishing and international collaboration are helping to bridge this gap, leading to increased global visibility of research from these regions.

## Conclusions

Mitochondria are essential organelles for many aspects of cellular homeostasis as well as critical regulators of cell death. Alterations in mitochondrial function not only impact cellular metabolism but also critically influence whole-body health leading to disease development and progression, particularly cancer which is a major cause of death worldwide. Based on our bibliometric analyses, a total of 169,555 publications were identified in PubMed relating to ‘mitochondria’, of which 34,949 (20.61%) concerned ‘mitochondria’ and ‘dysfunction’ and 22,406 (13.21%) addressed ‘mitochondria’ and ‘cancer’. It was therefore concluded that not all mitochondrial dysfunctions lead to cancer or enhance its progression.

The USA occupied the first position among countries contributing the highest number of publications (5695 articles), followed by China (4660 articles), Italy (1200 articles), and Japan (1144 articles). The USA and China are the leaders in this field, whereas Egypt came in the thirty-eighth position with 84 publications (0.46%), which may be due to the extremely limited research and development funding in Egypt, causing scientists to focus mainly on local journals that do not appear in PubMed or WoS.

A detailed analysis of the link between mitochondrial research and liver cancer shows that this research is still very basic, focused on a few countries and researchers, and without any real collaboration. It would be interesting to know whether this pattern is specific to liver cancer, or whether the same results apply to other types of cancers.

## Supplementary Information

Additional file1 (DOCX 25 KB)

## Data Availability

All data generated and analyzed in this study are included in this article.
