# Supplementary material for: Mitochondria in cancer: a comprehensive review, bibliometric analysis, and future perspectives
Source: Discov Oncol. 2025 Apr 11;16:517. doi: 10.1007/s12672-025-02139-5 (PMC11992316; doi:10.1007/s12672-025-02139-5)
Supplement: Supplementary file 1 — Additional file1 (DOCX 25 KB) [file 12672_2025_2139_MOESM1_ESM.docx]

**Table S1:** Two queries string in the period 1990 – 2023 to identify the publications relating to mitochondria and cancer.

| **Number** | **Query string** |
| --- | --- |
| **Query 1** | mitochondria [MeSH Terms] OR mitochondria [ti] AND (Journal Article [Ptyp] OR Review [Ptyp]) |
| **Query 2** | Neoplasms [MeSH Terms] OR Cancer [Title] OR Cancer [Other Term] OR Tumor [Title] OR Tumor [Other Term] OR clin bull [Journal] OR gan no rinsho [Journal] OR z krebsforsch klin onkol cancer res clin oncol [Journal] OR z krebsforsch [Journal] OR cancer treat rev [Journal] OR j natl cancer inst [Journal] OR bull assoc fr etud cancer [Journal] OR nihon gan chiryo gakkai shi [Journal] OR rev inst nac cancerol mex [Journal] OR cancer biochem biophys [Journal] OR med pediatr oncol [Journal] OR br j cancer suppl [Journal] OR osterr z onkol [Journal] OR natl cancer inst res rep [Journal] OR clin oncol [Journal] OR osterr z erforsch bekampf krebskr [Journal] OR cancer lett [Journal] OR int j radiat oncol biol phys [Journal] OR cancer chemother rep [Journal] OR cancer treat rep [Journal] OR proc natl cancer conf [Journal] OR curr probl cancer [Journal] OR carcinog compr surv [Journal] OR cancer detect prev [Journal] OR leuk res [Journal] OR j dermatol surg oncol [Journal] OR cancer nurs [Journal] OR acta radiol oncol radiat phys biol [Journal] OR cancer chemother pharmacol [Journal] OR int adv surg oncol [Journal] OR onkologie [Journal] OR oncol nurs forum [Journal] OR gan to kagaku ryoho [Journal] OR j cancer res clin oncol [Journal] OR iarc monogr eval carcinog risk chem hum [Journal] OR nutr cancer [Journal] OR cancer clin trials [Journal] OR am j pediatr hematol oncol [Journal] OR head neck surg [Journal] OR cancer genet cytogenet [Journal] OR zhonghua zhong liu za zhi [Journal] OR iarc monogr eval carcinog risk chem hum suppl [Journal] OR carcinogenesis [Journal] OR cancer treat res [Journal] OR iarc sci publ [Journal] OR eur j gynaecol oncol [Journal] OR oncodev biol med [Journal] OR teratog carcinog mutagen [Journal] OR anticancer res [Journal] OR breast cancer res treat [Journal] OR eur j cancer clin oncol [Journal] OR invasion metastasis [Journal] OR am j clin oncol [Journal] OR acta radiol oncol [Journal] OR cancer surv [Journal] OR j biol response mod [Journal] OR med pediatr oncol suppl [Journal] OR cancer invest [Journal] OR hematol oncol [Journal] OR j exp clin cancer res [Journal] OR j clin oncol [Journal] OR j neurooncol [Journal] OR j psychosoc oncol [Journal] OR chemioterapia [Journal] OR med oncol tumor pharmacother [Journal] OR eksp onkol [Journal] OR radiother oncol [Journal] OR tumour biol [Journal] OR clin exp metastasis [Journal] OR j assoc pediatr oncol nurses [Journal] OR radiat med [Journal] OR j environ pathol toxicol oncol [Journal] OR semin surg oncol [Journal] OR eur j surg oncol [Journal] OR semin oncol nurs [Journal] OR important adv oncol [Journal] OR g ital oncol [Journal] OR int j hyperthermia [Journal] OR jpn j cancer res [Journal] OR adv immun cancer ther [Journal] OR strahlenther onkol [Journal] OR cancer metastasis rev [Journal] OR cancer immunol immunother [Journal] OR dimens oncol nurs [Journal] OR j cancer educ [Journal] OR nci monogr [Journal] OR pediatr hematol oncol [Journal] OR sonderb strahlenther onkol [Journal] OR leukemia [Journal] OR acta oncol [Journal] OR hematol oncol clin north am [Journal] OR int j cancer suppl [Journal] OR oncogene [Journal] OR oncology williston park [Journal] OR int j biol markers [Journal] OR lung cancer [Journal] OR in vivo [Journal] OR neurofibromatosis [Journal] OR cancer detect prev suppl [Journal] OR mol carcinog [Journal] OR cancer chemother biol response modif [Journal] OR head neck [Journal] OR mol biother [Journal] OR ons news [Journal] OR suppl j med oncol tumor pharmacother [Journal] OR iarc monogr eval carcinog risks hum suppl [Journal] OR iarc monogr eval carcinog risks hum [Journal] OR sel cancer ther [Journal] OR j surg oncol suppl [Journal] OR crit rev oncog [Journal] OR crit rev oncol hematol [Journal] OR cancer commun [Journal] OR j pediatr oncol nurs [Journal] OR cancer cells [Journal] OR clin oncol r coll radiol [Journal] OR bull cancer radiother [Journal] OR eur j cancer [Journal] OR curr opin oncol [Journal] OR genes chromosomes cancer [Journal] OR leuk lymphoma [Journal] OR ann oncol [Journal] OR semin cancer biol [Journal] OR j natl cancer inst monogr [Journal] OR cancer causes control [Journal] OR j immunother 1991 [Journal] OR melanoma res [Journal] OR int j gynecol cancer [Journal] OR cancer epidemiol biomarkers prev [Journal] OR semin radiat oncol [Journal] OR oncol res [Journal] OR surg oncol [Journal] OR surg oncol clin n am [Journal] OR breast [Journal] OR eur j cancer b oral oncol [Journal] OR psychooncology [Journal] OR can oncol nurs j [Journal] OR eur j cancer prev [Journal] OR princess takamatsu symp [Journal] OR eur j cancer care engl [Journal] OR support care cancer [Journal] OR j oncol manag [Journal] OR can j oncol [Journal] OR int j oncol [Journal] OR j infus chemother [Journal] OR sonderb z strahlenther onkol [Journal] OR cancer pract [Journal] OR magy onkol [Journal] OR cancer biother [Journal] OR noshuyo byori [Journal] OR radiol oncol [Journal] OR j immunother emphasis tumor immunol [Journal] OR ann surg oncol [Journal] OR oncol rep [Journal] OR j egypt natl canc inst [Journal] OR ai zheng [Journal] OR klin onkol [Journal] OR rev mex cir ginecol cancer [Journal] OR cancer gene ther [Journal] OR med oncol [Journal] OR endocr relat cancer [Journal] OR radiat oncol investig [Journal] OR cancer control [Journal] OR oncologica [Journal] OR clin cancer res [Journal] OR curr oncol [Journal] OR breast j [Journal] OR j pediatr hematol oncol [Journal] OR j oncol pharm pract [Journal] OR cancer j sci am [Journal] OR semin urol oncol [Journal] OR j mammary gland biol neoplasia [Journal] OR j exp ther oncol [Journal] OR cancer biother radiopharm [Journal] OR oncologist [Journal] OR int j clin oncol [Journal] OR clin j oncol nurs [Journal] OR j immunother [Journal] OR pathol oncol res [Journal] OR oral oncol [Journal] OR cancer prev control [Journal] OR cancer radiother [Journal] OR brain tumor pathol [Journal] OR j registry manag [Journal] OR urol oncol [Journal] OR biochim biophys acta rev cancer [Journal] OR prostate cancer prostatic dis [Journal] OR j buon [Journal] OR eur j oncol nurs [Journal] OR gastric cancer [Journal] OR neoplasia [Journal] OR neuro oncol [Journal] OR breast cancer [Journal] OR curr oncol rep [Journal] OR clin lung cancer [Journal] OR fam cancer [Journal] OR clin breast cancer [Journal] OR clin lymphoma [Journal] OR curr treat options oncol [Journal] OR breast cancer res [Journal] OR cancer j [Journal] OR lancet oncol [Journal] OR bmc cancer [Journal] OR curr cancer drug targets [Journal] OR cancer immun [Journal] OR clin colorectal cancer [Journal] OR expert rev anticancer ther [Journal] OR nat rev cancer [Journal] OR zhongguo fei ai za zhi [Journal] OR integr cancer ther [Journal] OR cancer cell [Journal] OR asian pac j cancer prev [Journal] OR int j gastrointest cancer [Journal] OR cancer biol ther [Journal] OR technol cancer res treat [Journal] OR j cancer epidemiol prev [Journal] OR mol cancer [Journal] OR mol cancer res [Journal] OR suppl tumori [Journal] OR cancer res treat [Journal] OR clin prostate cancer [Journal] OR rep carcinog [Journal] OR j natl compr canc netw [Journal] OR clin adv hematol oncol [Journal] OR cancer sci [Journal] OR world j surg oncol [Journal] OR cancer imaging [Journal] OR j support oncol [Journal] OR vet comp oncol [Journal] OR pediatr blood cancer [Journal] OR cancer genomics proteomics [Journal] OR esophagus [Journal] OR cell oncol [Journal] OR nat clin pract oncol [Journal] OR exp oncol [Journal] OR am soc clin oncol educ book [Journal] OR asia pac j clin oncol [Journal] OR clin transl oncol [Journal] OR j cancer res ther [Journal] OR clin lymphoma myeloma [Journal] OR cancer biomark [Journal] OR future oncol [Journal] OR clin genitourin cancer [Journal] OR j oncol pract [Journal] OR j soc integr oncol [Journal] OR curr hematol malig rep [Journal] OR radiat oncol [Journal] OR recent pat anticancer drug discov [Journal] OR target oncol [Journal] OR j thorac oncol [Journal] OR ons connect [Journal] OR head neck pathol [Journal] OR j cancer surviv [Journal] OR mol oncol [Journal] OR pigment cell melanoma res [Journal] OR hematol oncol stem cell ther [Journal] OR j hematol oncol [Journal] OR j med imaging radiat oncol [Journal] OR cancer prev res phila [Journal] OR j gastrointest cancer [Journal] OR head neck oncol [Journal] OR j gynecol oncol [Journal] OR chin j cancer [Journal] OR cancer cytopathol [Journal] OR nat rev clin oncol [Journal] OR gulf j oncolog [Journal] OR cancer epidemiol [Journal] OR horm cancer [Journal] OR rep carcinog backgr doc [Journal] OR clin lymphoma myeloma leuk [Journal] OR thorac cancer [Journal] OR oncotarget [Journal] OR j geriatr oncol [Journal] OR cancer genet [Journal] OR anal cell pathol amst [Journal] OR j adolesc young adult oncol [Journal] OR j cachexia sarcopenia muscle [Journal] OR cell oncol dordr [Journal] OR pract radiat oncol [Journal] OR cancer discov [Journal] OR int j surg oncol [Journal] OR blood cancer j [Journal] OR oncoimmunology [Journal] OR cancer biol med [Journal] OR cns oncol [Journal] OR cancer med [Journal] OR eur thyroid j [Journal] OR chin clin oncol [Journal] OR cancer immunol res [Journal] OR j immunother cancer [Journal] OR j community support oncol [Journal] OR oncol res treat [Journal] OR prog tumor res [Journal] OR rep carcinog monogr [Journal] OR j cancer policy [Journal] OR lancet haematol [Journal] OR jama oncol [Journal] OR papillomavirus res [Journal] OR trends cancer [Journal] OR j glob oncol [Journal] OR cancer treat res commun [Journal] OR jco precis oncol [Journal] OR jco clin cancer inform [Journal] OR jnci cancer spectr [Journal] OR cancer commun lond [Journal] OR eur urol oncol [Journal] OR cancer rep hoboken [Journal] OR jco oncol pract [Journal] OR jco glob oncol [Journal] OR nat cancer [Journal] OR blood cancer discov [Journal] OR radiol imaging cancer [Journal] OR tumour virus res [Journal] OR prog clin cancer [Journal] OR rev esp oncol [Journal] OR proc can cancer conf [Journal] OR cancer res [Journal] |

**Table S2:** Queries string to illustrate the evolution of the top four cancer/neoplasm types namely liver, brain, lung, and breast displaying the highest numbers of published articles worldwide in the period of 1990 – 2023.

| **MeSH terms** | **Queries string** |
| --- | --- |
| **Mitochondria** | Mitochondria [MeSH Terms] OR mitochondria [ti]) AND Journal Article [ptyp] OR Review [Ptyp] |
| **Liver Neoplasms** | Liver Neoplasms [Mesh Terms] OR Carcinoma, Hepatocellular [Mesh Terms] OR (hepatic cancer[ti]) |
| **Brain Neoplasms** | Brain Neoplasms [Mesh Terms] OR Glioma [Mesh Terms] OR Glioblastoma [Mesh Terms] OR Neuroblastoma [Mesh Terms] OR Meningeal Neoplasms [Mesh Terms] OR Meningioma [Mesh Terms] OR pituitary neoplasms [Mesh Terms] OR Brain Neoplasms [ti] OR Glioma [ti] OR Glioblastoma [ti] OR Neuroblastoma [ti] OR Meningeal Neoplasms [ti] OR Meningioma [ti]) |
| **Lung Neoplasms** | Lung Neoplasms [Mesh Terms] OR Carcinoma, Non-Small-Cell Lung [Mesh Terms] OR Mesothelioma [Mesh Terms] OR Pleural Neoplasms [Mesh Terms] OR (Lung [ti] AND cancer [ti]) OR (Lung Neoplasm s[ti] OR Carcinoma, Non-Small-Cell Lung [ti] OR Mesothelioma [ti] OR Pleural Neoplasms [ti]) |
| **Breast Neoplasms** | (Breast Neoplasms [Mesh Terms] OR Carcinoma, Ductal, Breast [Mesh Terms]) OR (breast [ti] AND Cancer[ti]) |

**Table S3:** The overall number of Web of Science documents in each scientific categories related mitochondria to liver neoplasms as well as the numbers that are in the top 1% and 10% most cited articles within the study period of 1990 – 2023.

| **Categories** | **Web of Science documents** | **Percent** | **Category Normalized Citation Impact** | **Documents in Top 1%** | **Documents in Top 10%** |
| --- | --- | --- | --- | --- | --- |
| **Baseline for All Items** | **1231** |  | **1.32** | **14** | **215** |
| Biochemistry & Molecular Biology | 356 | 28.9 | 1.04 | 1 | 40 |
| Oncology | 287 | 23.3 | 1.19 | 1 | 45 |
| Cell Biology | 221 | 18.0 | 1.08 | 1 | 30 |
| Pharmacology & Pharmacy | 176 | 14.3 | 1.48 | 0 | 38 |
| Medicine, Research & Experimental | 109 | 8.9 | 1.25 | 0 | 14 |
| Toxicology | 86 | 7.0 | 1.68 | 2 | 18 |
| Gastroenterology & Hepatology | 82 | 6.7 | 2.04 | 4 | 18 |
| Biophysics | 81 | 6.6 | 1.11 | 0 | 8 |
| Genetics & Heredity | 58 | 4.7 | 1.36 | 1 | 8 |
| Chemistry, Medicinal | 50 | 4.1 | 1.62 | 1 | 13 |
| Chemistry, Multidisciplinary | 49 | 4.0 | 1.08 | 0 | 4 |
| Endocrinology & Metabolism | 49 | 4.0 | 1.74 | 1 | 14 |
| Biotechnology & Applied Microbiology | 32 | 2.6 | 1.05 | 0 | 5 |
| Integrative & Complementary Medicine | 28 | 2.3 | 1.46 | 0 | 5 |
| Food Science & Technology | 25 | 2.0 | 1.92 | 0 | 7 |
| Physiology | 25 | 2.0 | 1.03 | 0 | 3 |
| Plant Sciences | 23 | 1.9 | 1.37 | 0 | 3 |
| Chemistry, Applied | 20 | 1.6 | 1.30 | 0 | 4 |
| Pathology | 17 | 1.4 | 0.97 | 0 | 1 |
| Biology | 15 | 1.2 | 1.37 | 0 | 2 |
| Nutrition & Dietetics | 14 | 1.1 | 0.94 | 0 | 2 |
| Virology | 14 | 1.1 | 1.76 | 1 | 4 |
| Immunology | 12 | 1.0 | 1.17 | 0 | 2 |
| Surgery | 11 | 0.9 | 0.71 | 0 | 1 |
| Environmental Sciences | 10 | 0.8 | 1.66 | 1 | 1 |
| Agriculture, Multidisciplinary | 9 | 0.7 | 2.85 | 0 | 3 |
| Medical Laboratory Technology | 9 | 0.7 | 1.92 | 0 | 3 |
| Microbiology | 9 | 0.7 | 1.05 | 0 | 1 |
| Nanoscience & Nanotechnology | 9 | 0.7 | 1.10 | 0 | 1 |
| Biochemical Research Methods | 8 | 0.6 | 0.80 | 0 | 0 |
